# Therapeutical and Pharmaceutical Notes

**Published:** 1900-04

**Authors:** W. J. Martin

**Affiliations:** Kankakee, Ill.


### THERAPEUTICAL AND PHARMACEUTICAL NOTES.

UNDER THE DIRECTION OF W. J. MARTIN, M.D.C.,
KANKAKEE, ILL.

SUTURE-WOUNDS OF THE HEART. Elsburg (*Journal of Experimental Medicine*, vol. iv., Nos. 5 and 6), as a result of a historical and experimental study, states that suture-wound of the heart, as a final result, is an operation worthy of consideration in some cases and often justifiable; that this procedure in animals and also in man is devoid of the danger of a sudden arrest of motion due to manipulation, unless Kronecker's coördination centre be injured. The suture should be an interrupted one of silk, applied in most cases so that the epicardium and superficial layer of the myocardium are the only ones penetrated, and tied when possible during diastole. The laboratory experiments on which these conclusions are based are given in detail.—*Therapeutic Gazette.*

THE IMMEDIATE HERMETIC SEALING OF WOUNDS. Marcy (*Columbus Medical Journal*, December 5, 1899), after a preliminary discussion of this subject, states that the object of the surgeon should be to maintain a wound aseptic. Like structures should be coaptated and held at rest by buried aseptic, absorbable sutures, and the technique is completed by the application of a dressing to prevent subsequent infection. This is secured so simply and easily by iodoform collodion, strengthened by a few fibres of cotton, that this dressing reaches an ideal completion. It is fluid-proof, in that no exudate can escape from beneath it, and as a consequence it is germ-proof, in that by no means is it possible that any foreign material can enter the wound. Beyond this it holds in even coaptation and at rest with a certain fixity of support the approximated parts. Iodoform is soluble in it, and under certain conditions it is inhibitory to the development of micrococcus albus in the proliferating epithelium, and for this purpose is quite as valuable as the silver salts, which are much more difficult of application. To one who may doubt the effect of a potent agent seemingly locked up in a collodion film, we need only to cite the powerful desiccating effect of cantharidal collodion. A wound made and maintained aseptic in well-vitalized structures, held at rest by buried tendon sutures and sealed with iodoform collodion, will not suppurate, and will be followed by a non-inflammatory primary union.

The fear, the anxiety, the constant supervision and watchfulness of the wound by nurse and attendant are entirely abrogated; subsequent dressing is of no avail except to keep the parts from extraneous injury. The work of the surgeon for good or ill has its finality at the period of manipulative intervention.—*Ibid.*

POTASSIUM CHLORATE FOR BURNS. The immediate application of a cold saturated solution of potassium chlorate has an excellent effect on burns, relieving the pain rapidly. Superficial burns may be permanently dressed with a solution of the salt, but deeper wounds should be treated with full antiseptic precaution.—*Semaine Med.*

QUININE AND THE MALARIAL PARASITE. Lomonaco and Panichi (*Centralblatt für die medicinischen Wissenschaften*, August 19, 1899, No. 33) have studied with direct microscopical observation the effects of quinine on the blood of malarial patients (quartan type) who had not been treated with quinine. In the case of the early form of the parasite, when a drop of an isotonic (0.9 NaCl) and at the same time isoviscous (2 per cent. gum) solution was passed under the cover slip, the red corpuscles at once became mobile, collided with one another, and became altered in shape. One corpuscle, which moved least, and therefore retained its shape best, was especially studied. The intracorpuscular parasite displayed active amœboid movements, throwing out pseudopodia, and becoming more slender. This lasted only for a few seconds, when the parasite assumed an elliptical shape, with its pigment granules arranged peripherally. This resting stage did not continue long, for movements soon became apparent, and could still be made out some hours later. If, however, instead of the aforesaid solution, a drop of watery quinine solution (1 in 1500) was added, contraction of the parasite occurred, accompanied by tremulous movements of the pseudopodia, with central arrangements of the pigment granules. At the end of a quarter of an hour the parasite recovered its shape and movements (pseudopodia), the pigment becoming arranged peripherally. In the slightly more developed phase, when about two-fifths of the red corpusles are occupied by the parasite, quinine gave rise to the following phenomena: The parasite contracted, drew in its pseudopodia, and became more or less spherical. The granules, situated chiefly at the periphery, took on active movements. Gradually the parasite became quite spherical, and all the pigment granules peripheral. Once more did the latter take on active movements, the parasite appeared more refractile, and gradually worked its way out of the corpuscle, to the margin of which it remained attached. This phenomenon is independent of the currents in the fluid; indeed, it may occur in a direction contrary to these. When the currents were marked the red corpuscle was carried along with the parasite glued to it. Later the corpuscle lost its red color, becoming ghost-like; and the parasite, now free and retaining its shape, exhibited active movements of its peripherally placed granules. The decolorization of the normal red corpuscles did not occur when a few drops of a 9 per cent. salt solution was added to the quinine solution, but the parasite-containing corpuscle did. In isotonic and isoviscous salt solution, with or without quinine, the parasites in a late stage of development exhibited active movements, but never worked their way out of the corpuscles.

Marchiafava and Celli (*Archiv per la Scienze Medica*, vol. x., p. 193) have noted, without laying much stress upon the point, that after the exhibition of quinine most of the parasites were motionless, and a few were observed in the act of leaving the corpuscle. Lomonaco and Panichi conclude from further experiments that the diapedesis of the parasite depends upon the latter's vitality; that it is an active phenomenon. As the red blood-corpuscle is necessary for the life of the parasite, the action of quinine can be very simply explained, namely, quinine, by driving the parasite out of its element, places it under conditions unfavorable and destructive to its development. A practical point as to treatment, therefore, follows, namely, that quinine should not be given in the febrile stage of the disease, but during the non-febrile intervals, when the early forms of the parasites are present in the blood in the greatest number.—*British Medical Journal*, October 21, 1899.

CAMPHOROXOL. A disinfectant vulnerary, composed of 38 parts of alcohol and 68 parts of a 1 per cent. solution of camphor in a 3 per cent. solution of hydrogen dioxide.

